# Bilateral globus pallidus interna deep brain stimulation in the treatment of mixed cerebral palsy in ataxia with dyskinesia: a case report

**DOI:** 10.3389/fneur.2023.1238292

**Published:** 2023-08-10

**Authors:** Lei Chang, Bei Luo, Wenwen Dong, Chang Qiu, Yue Lu, Jian Sun, Jiuqi Yan, Wenbin Zhang, Jun Yan

**Affiliations:** ^1^Department of Functional Neurosurgery, The Affiliated Brain Hospital of Nanjing Medical University, Nanjing, China; ^2^Department of Geriatric Neurology, The Affiliated Brain Hospital of Nanjing Medical University, Nanjing, China

**Keywords:** mixed cerebral palsy, deep brain stimulation, globus pallidus interna, neuromodulation, neurodevelopment

## Abstract

**Background:**

Cerebral palsy (CP), a complex syndrome with multiple etiologies, is characterized by a range of movement disorders within the hypokinetic and hyperkinetic spectrum (dystonia or choreoathetosis). CP is often accompanied by neurological and psychiatric signs, such as spasticity, ataxia, and cognitive disorders. Although current treatment options for CP include pharmacological interventions, rehabilitation programs, and spasticity relief surgery, their effectiveness remains limited. Deep brain stimulation (DBS) has demonstrated significant effectiveness in managing dyskinesia; however, its potential therapeutic effect on CP remains determined.

**Methods:**

We present a case of a 44-year-old Asian female who was born as a twin with neonatal ischemic–hypoxic encephalopathy due to prolonged labor and delivery. She was diagnosed with CP at the age of 1 year. The patient exhibited delayed development compared to her peers and presented with various symptoms, including slurred speech, broad-based gait, horseshoe inversion of the right lower extremity, involuntary shaking of the upper extremities bilaterally, and hypotonia and showed no improvement with levodopa therapy. Two years ago, she developed progressive head tremors, which worsened during periods of tension and improved during sleep. As medical treatments proved ineffective and there were no contraindications to surgery, we performed bilateral globus pallidus interna DBS (GPi-DBS) to alleviate her motor dysfunction.

**Results:**

Following a 6-month follow-up, the patient demonstrated significant improvements in motor symptoms, including head and limb tremors and dystonia. In addition, significant improvement was observed in her overall psychological well-being, as evidenced by reduced anxiety and depression levels.

**Conclusion:**

DBS is an effective treatment for dyskinesia symptoms associated with CP in adults. Moreover, its effectiveness may continue to increase over time.

## 1. Introduction

CP is a group of persistent central motor and postural developmental disorders that result from non-progressive brain damage in the developing fetus or infant, leading to limited movement syndromes. The clinical manifestations of CP are diverse but generally include delayed motor development with reduced active movement, dystonia, abnormal posture, and abnormal reflexes. In addition, it is often accompanied by sensory, cognitive, and behavioral disorders (1). Currently, no targeted pharmacological interventions are available to treat CP; therefore, rehabilitation therapy remains the primary treatment option. Traditional surgical interventions in CP aimed at improving rehabilitation outcomes by reducing spasticity and deformities. However, novel therapeutic interventions are needed to further enhance the quality of life in these individuals. This report presents a rare case of DBS used to treat mixed cerebral palsy characterized by ataxia and dyskinesia. The successful application of DBS in this case offers a fresh perspective and potential breakthrough in the treatment of CP.

## 2. Methods and results

We present a case of a 44-year-old Asian female with a 43-year medical history. She was born as a twin with neonatal ischemic–hypoxic encephalopathy due to prolonged labor and delivery. She was diagnosed with CP at the age of 1 year. The patient experienced delayed speech and physical development compared to her peers, which led to unclear speech, unsteady walking, broad-based gait, horseshoe inversion of the right lower extremity, involuntary shaking of the upper extremities bilaterally, hypotonia, and no improvement with levodopa therapy. During the interview, the patient denied any history of hyperbilirubinemia at birth and drug poisoning or overdose, effectively ruling out kernicterus and toxic or drug-induced tremors. Laboratory tests confirmed that the patient’s thyroxine levels were within the normal range, ruling out hyperthyroidism. In addition, imaging results revealed a stable, slightly lower signal in the frontal lobe, providing further evidence supporting the diagnosis of CP. Two years ago, the patient started experiencing involuntary head tremors, exacerbated by tension and emotional excitement but improved during sleep. The tremors were not accompained by limb stiffness and slow movement. Over the past 10 days, the patient experienced worsening of the involuntary head shaking, with no significant changes observed in her imaging data compared to previous scans. Upon admission, the physician prescribed Madopar Dispersible tablet, anisodine, hydrochloric-acid arotinolol to improve limb tremors, idebenone tablet to improve mitochondrial function, and aceglutamide injection to nourish the nerves. Madopar is a combination of levodopa and benserazide. Levodopa, the primary component, is a dopamine precursor that crosses the blood–brain barrier to produce central dopaminergic effects; it is primarily used in Parkinson’s disease and dopa-responsive dystonia (2, 3). Anisodine exhibits central anticholinergic effects by hindering acetylcholine and M choline receptor binding, thereby blocking nerve impulse transmission and interfering with physiological functions mediated by cholinergic neurotransmission. Anisodine manages tremors caused by nervous system inflammation and exhibits neuroprotective effects when cerebral perfusion is insufficient (4). Hydrochloric acid arotinolol acts as a nonselective β-adrenergic antagonist and is a second-line recommendation for tremor management (5, 6). Idebenone, a synthetic analog of coenzyme Q10, is used to treat cerebrovascular disease-related impairment of brain function and psychobehavioral disorders. Multiple studies have demonstrated its therapeutic effect in patients with ataxia, leading to improvements in gait, posture, and motor function to varying degrees (7, 8). Although it lacks high-level evidence-based medicine support, physicians have explored its use for dystonia secondary to CP in a symptom-driven manner. Neurotrophic drugs are commonly used in CP management, with aceglutamide being an effective option for improving nerve cell metabolism and preserving nervous stress function, thereby contributing to the recovery of motor function (9). However, only neurotrophic drugs are retained after other treatments have proven ineffective. These treatments did not yield the desired effect, as evidenced by relevant assessments conducted upon admission (Table 1) (10). As a result, on July 12, 2022, GPi-DBS was performed under general anesthesia to alleviate her motor dysfunction.

**Table 1:** Pre- and post-operative clinical scores following GPi-DBS in the patient with CP.

Scales	Subscale	Pre-op	1 months post-op	3 months post-op	6 months post-op	Improvement rate (%)
HAMA		8.00	7.00	7.00	6.00	25.00
HAMD		9.00	9.00	8.00	8.00	11.11
MMSE		28.00	28.00	28.00	28.00	0
MoCA		23.00	23.00	23.00	23.00	0
BFMDRS	Movement scores	54.00	50.00	36.00	35.00	35.19	Disability scores	15.00	15.00	13.00	12.00	20.00
FTM-TRS		84.00	74.00	57.00	49.00	41.67
SF-36	Physical functioning	25.00	25.00	40.00	45.00		Role limitation (physical function)	0	25.00	25.00	50.00		Body pain	100.00	100.00	100.00	100.00		General health	15.00	15.00	20.00	20.00		Vitality	45.00	50.00	50.00	55.00		Social functioning	25.00	25.00	50.00	62.50		Role limitation (emotional)	0	33.33	66.67	66.67		Mental health	36.00	36.00	40.00	65.00		Total scores	246.00	309.33	391.67	464.17	47.00

HAMA, Hamilton Anxiety Scale; HAMD, Hamilton Depression Rating Scale; BFMDRS, Burke-Fahn-Marsden Dystonia Rating Scale; FTM-TRS, Fahn-Tolosa-Marin Tremor Rating Scale; SF-36, 36-item Short Form Health Survey.

**Figure 1:**
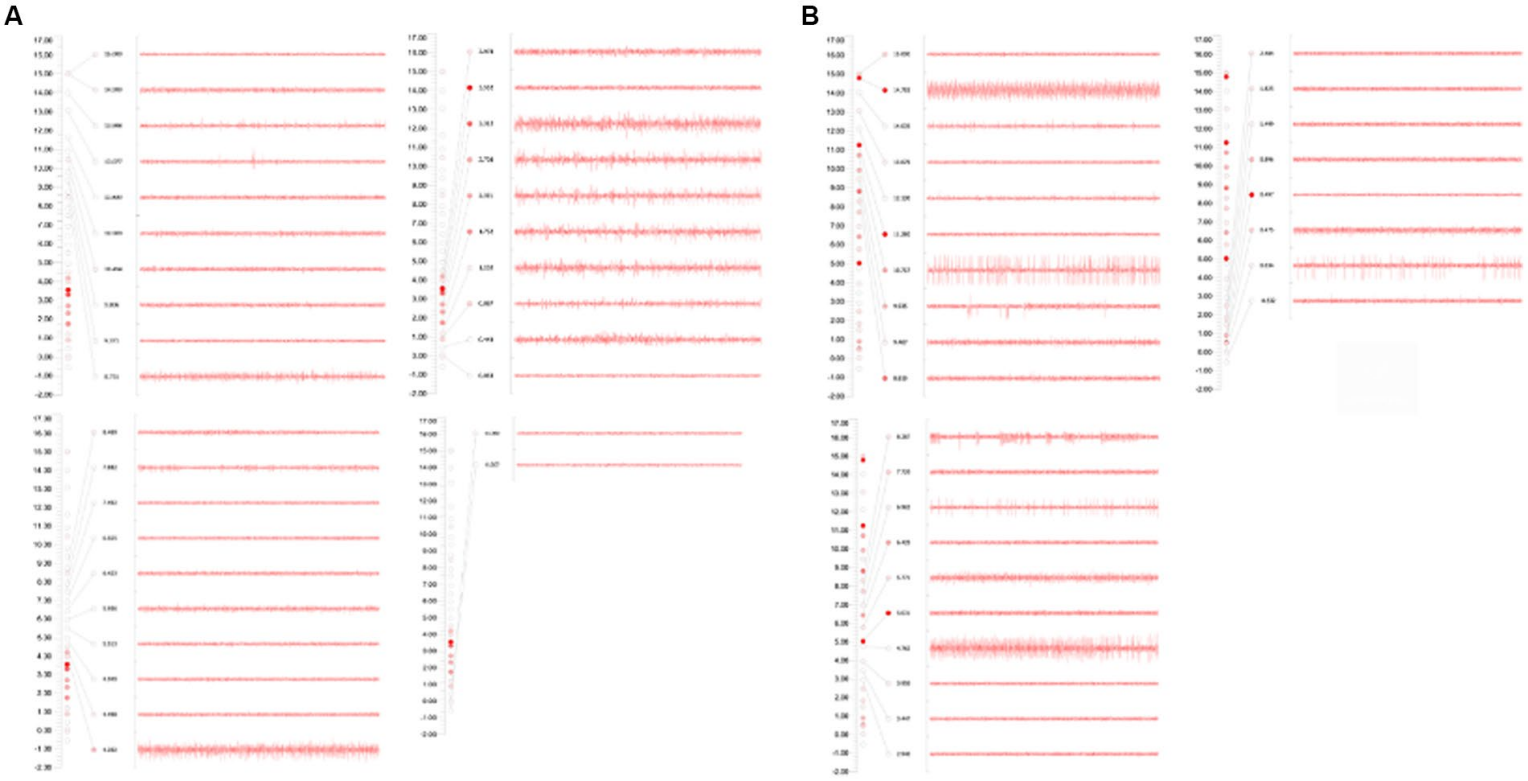
**(A)** GPi on the left side: during the monitoring process, GPi discharge was recorded from 6.50 mm above the target to 0.50 mm above the target, and the electrode was placed at 0.50 mm above the target. **(B)** GPi on the right side: during the monitoring process, GPi discharge was recorded from 6.20 mm above the target to 0.30 mm above the target; and the electrode was placed 0.50 mm above the target.

**Figure 2:**
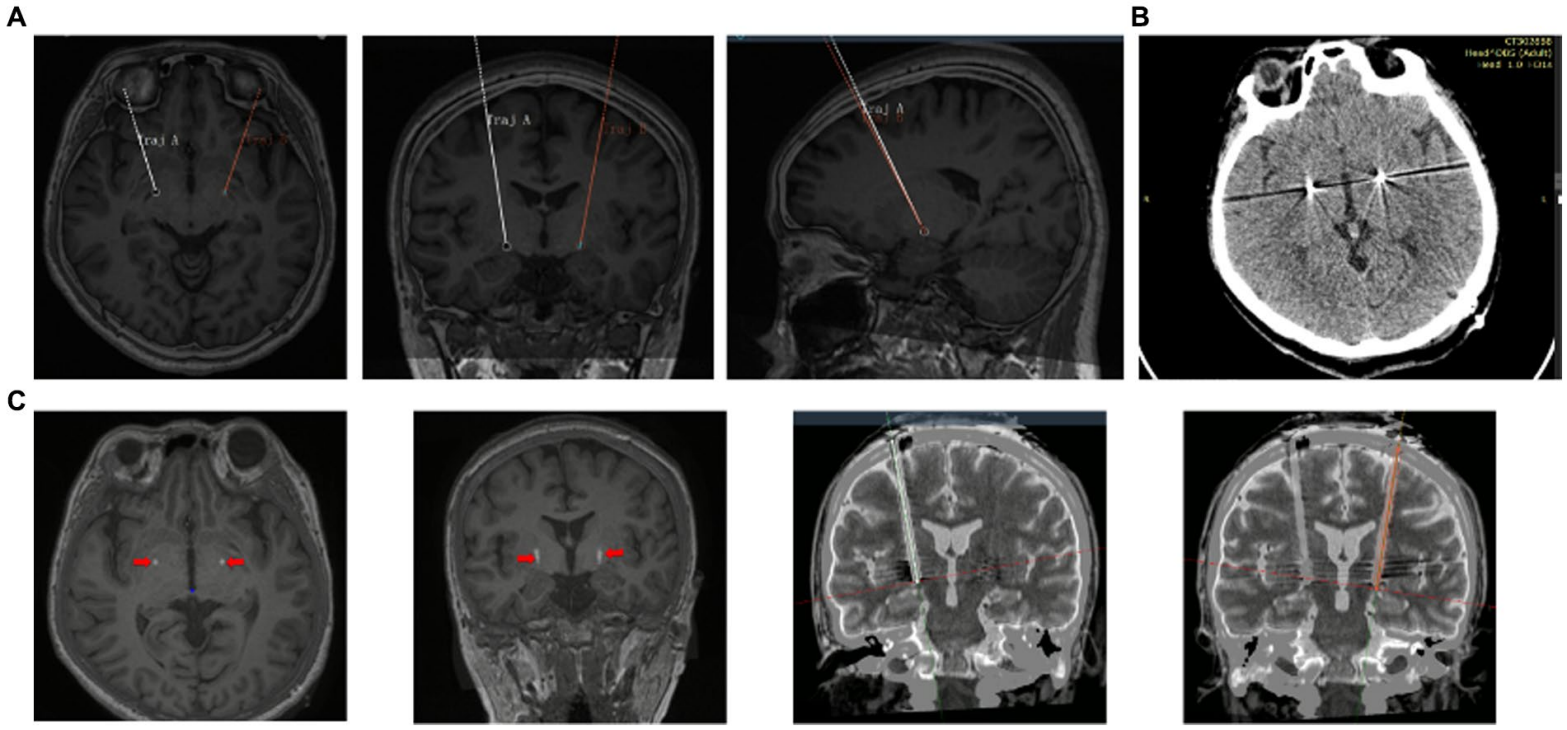
**(A)** Preoperative craniocerebral 3.0 T MRI and skull CT data with frame were imported into Surgiplan software to develop the surgical plan. **(B)** CT scan after operation showed no bleeding or bubble around the electrodes. **(C)** Postoperative CT and preoperative MRI fusion showed that electrode implantation trajectory and implantation position were accurate.

The patient underwent bilateral placement of DBS leads (L302, Beijing PINS Medical Co., Ltd., Beijing, China) targeting the GPi. The surgical plan was developed using preoperative brain 3.0 T magnetic resonance imaging and CT brain data imported into SurgiPlan software (Elekta Instruments, Stockholm, Sweden). The frame coordinates of GPi were determined based on the scanning results, with the left coordinates being X = 116.5, Y = 94, Z = 109, arc = 103.5, ring = 54, and the right coordinates being X = 81, Y = 93.5, Z = 109, arc = 84, ring = 56. During the operation, an Omega electrophysiological instrument (Alpha Omega Engineering Ltd., Nazareth Illit, ISR) and a recording electrode were used to monitor the discharge of a single cell in the GPi nucleus to determine the depth of electrode implantation, with both the left and right sides being implanted 0.5 mm above the target. The accuracy of the electrode placement was confirmed through a fusion of preoperative and postoperative brain magnetic resonance imaging and computed tomography data, respectively (Figures 1, 2). No adverse events, such as paresthesia and dysarthria, occurred during the procedure.

**Figure 3:**
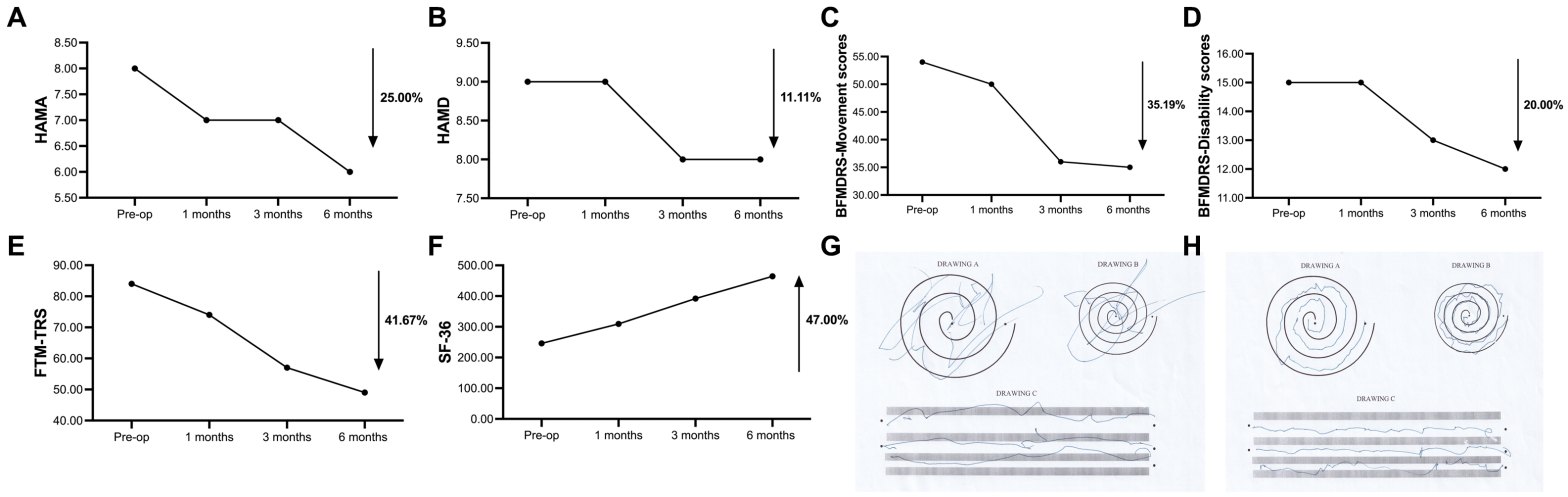
**(A)** Trends in HAMA scores. **(B)** Trends in HAMD scores. **(C)** Trends in BFMDRS-Movement scores. **(D)** Trends in BFMDRS-Disability scores. **(E)** Trends in FTM-TRS scores. **(F)** Trends in SF-36 scores. **(G,H)** Drawing A, B, C of FTM-TRS was tested before surgery and 6 months after surgery using the dominant and severely symptomatic right hand. HAMA, Hamilton Anxiety Scale; HAMD, Hamilton Depression Rating Scale; BFMDRS, Burke-Fahn-Marsden Dystonia Rating Scale; FTM-TRS, Fahn-Tolosa-Marin Tremor Rating Scale; SF-36, 36-item Short Form Health Survey.

To avoid the microlesion effect, the patient began using the device 2 weeks after surgery. The patient was followed up for 6 months postoperatively, and all programmed parameters and scale assessments are presented in (Figure 3; Tables 1, 2). Initially, we switched on the electrodes on both sides of the GPi and began with the following parameters: 2.0 V for voltage, 60 μs for pulse width, and 120 Hz for frequency. As we increased the frequency parameters to 150 Hz, the patient experienced a reduced head tremor amplitude. In functional brain diseases, voltage is directly related to the therapeutic effect. Although a wider pulse width is usually required for dystonia treatment, short pulse width stimulation can expand the upper limit of the therapeutic window. Therefore, finding the right balance in parameters is essential. Over the next 6 months, we gradually adjusted the parameters based on the patient’s condition, resulting in 3.15 V (R) and 3.65 V (L) for voltage, 70 μs (R) and 80 μs (L) for pulse width, and 155 Hz for frequency. These adjustments led to an overall improvement in the patient’s condition. Notably, head and left limb tremors improved significantly, whereas the right limb improved gradually but remained more pronounced in the writing test. Furthermore, the patient reported an expanded ability to perform daily activities and displayed optimism regarding her current and future prospects. Various assessment scores improved to different extents, including HAMA (25.00%), HAMD (11.11%), BFMDRS Movement scores (35.19%), BFMDRS Disability scores (20.00%), FTM-TRS (41.67%), and SF-36 (47.00%). The trend suggests that patients will benefit more from prolonged stimulation.

**Table 2:** Detailed parameters of program control during patient follow-up.

Time		Contact	Frequency (Hz)	Pulse width (μs)	Voltage (V)
2 weeks	Right	C + 2-	150	60	2.00	Left	C + 6-	150	60	2.00
1 months	Right	C + 2-	150	70	2.40	Left	C + 6-	150	80	3.00
3 months	Right	C + 2-	150	70	2.50	Left	C + 6-	150	80	3.10
6 months	Right	C + 2-	155	70	3.15	Left	C + 6-	155	80	3.65

## 3. Discussion

CP has a prevalence of approximately 0.1–0.5% and is caused by various factors, such as perinatal asphyxia, kernicterus, and metabolic brain injury (11). The hallmark of CP is motor control disorder, resulting in abnormal movement and posture (12). Current treatments for CP primarily involve oral medications, but no high-quality clinical evidence exists to guide treatment. Although botulinum toxin and intrathecal baclofen may offer therapeutic benefits for CP symptoms, their use carries risks of respiratory depression and catheter failure. In addition, the efficacy of selective posterior rhizotomy, a destructive surgical intervention, is limited and irreversible, and its efficacy is debated. As a relatively conservative surgical approach, DBS is increasingly recognized as a safe and effective treatment for refractory CP, with stimulation parameters tailored by the surgeon based on individual patient symptoms. The primary DBS targets are the ventral intermediate nucleus (Vim) and GPi. Previous comparative studies have demonstrated that GPi-DBS is more effective for patients with acquired dystonia, tremor, and without spasticity, with a more pronounced effect over time, whereas Vim-DBS is more sensitive to rapid movement disorders (13, 14). Several small sample-size studies have reported that 13 patients showed significant improvement following GPi-DBS. This improvement was evident through an average improvement of 24% in BFMDRS Movement scores. Moreover, the positive effects of GPi-DBS continue over time, with only one patient failing to respond to GPi and Vim stimulation, consistent with previous studies (15, 16). Based on patient needs and a literature review, we selected GPi as the implantation target. Recent evidence indicates connections between cerebellar and basal ganglia areas, including dense projections between the cerebellum and striatum and attenuation of abnormal oscillatory activity of cerebellar nuclei relieving tremors (17, 18). The cerebellum and basal ganglia are interconnected through red cortical connections and nonsynaptic pathways in the thalamus. Additionally, research studies have indicated that the cerebellum is often protected from damage in hypoxic encephalopathy (19, 20). The cerebellum is now being considered as a potential target for stimulation, particularly the dentato-rubro-thalamic tract, which could be beneficial for patients with refractory dystonia and spasticity (21, 22).

Although invasive neuromodulation is considered a “last resort” treatment for CP, research in this area is still in its early stages, consisting mainly of individual cases and small sample-size studies. In addition, determining appropriate patient screening criteria remains challenging due to the heterogeneity of CP symptomology. These factors have contributed to slow research progress and a lack of consensus on optimal stimulation targets and timing of DBS implantation. Although initial studies have demonstrated the efficacy of CP-DBS for adults, it is unclear whether early DBS intervention is necessary for children with CP. Early treatment with DBS may facilitate neural remodeling, regulate adverse developmental outcomes, and prevent deformities; surgical adjustments may be necessary during puberty growth spurts.

In summary, bilateral GPi-DBS in this rare case of refractory mixed CP demonstrated a surprisingly positive therapeutic effect. This result highlights the feasibility and effectiveness of DBS for CP treatment, potentially involving the regulation of a highly integrated neural network comprising the cerebral cortex, cerebellum, and basal ganglia. It also emphasizes the importance for conducting large sample size and multi-center CP-DBS research.

## Data availability statement

The original contributions presented in the study are included in the article/supplementary material, further inquiries can be directed to the corresponding authors.

## Ethics statement

The studies involving humans were approved by the ethics committee of Nanjing Brain Hospital Affiliated to Nanjing Medical University. The studies were conducted in accordance with the local legislation and institutional requirements. The participants provided their written informed consent to participate in this study. Written informed consent was obtained from the individual (s) for the publication of any potentially identifiable images or data included in this article.

## Author contributions

LC, BL, WD, CQ, YL, JS, JY, JY, and WZ contributed to the study conception and design. Data collection and analysis were performed by BL, CQ, and YL. The first draft of the manuscript was written by LC and WD. WZ edited and revised the manuscript. All authors contributed to the article and approved the submitted version.

## Funding

This study was supported by the grant from subtopic of the 13th Five-Year National Key Research and Development Plan (No. 2016YFC0105901NNZ), the Key R&D Program of Jiangsu Science and Technology Project (Nos. BE2022049 and BE2022049-1), and Nanjing Health Science and Technology Development Special Fund Project (No. ZKX20031).

## Conflict of interest

The authors declare that the research was conducted in the absence of any commercial or financial relationships that could be construed as a potential conflict of interest.

## Publisher’s note

All claims expressed in this article are solely those of the authors and do not necessarily represent those of their affiliated organizations, or those of the publisher, the editors and the reviewers. Any product that may be evaluated in this article, or claim that may be made by its manufacturer, is not guaranteed or endorsed by the publisher.

## Abbreviations

CP: cerebral palsy
DBS: deep brain stimulation
GPi: globus pallidus interna
Vim: ventral intermediate
DRT: dentato-rubro-thalamic tract
HAMA: Hamilton Anxiety Scale; HAMD, Hamilton Depression Rating Scale
BFMDRS: Burke-Fahn-Marsden Dystonia Rating Scale
FTM-TRS: Fahn-Tolosa-Marin Tremor Rating Scale
SF-36: 36-item short-form health survey

## References

[1] Koman LA Smith BP Shilt JS. Cerebral palsy. Lancet. (2004) 363:1619–31. doi: 10.1016/S0140-6736(04)16207-7 15145637

[2] Fehlings D Brown L Harvey A Himmelmann K Lin JP Macintosh A. Pharmacological and neurosurgical interventions for managing dystonia in cerebral palsy: a systematic review. Dev Med Child Neurol. (2018) 60:356–66. doi: 10.1111/dmcn.13652 29405267

[3] Masson R Pagliano E Baranello G. Efficacy of oral pharmacological treatments in dyskinetic cerebral palsy: a systematic review. Dev Med Child Neurol. (2017) 59:1237–48. doi: 10.1111/dmcn.13532, PMID: 28872668

[4] Chen D Peng C Xie X Chen Q Liu H Zhang S. Low dose of Anisodine Hydrobromide induced neuroprotective effects in chronic cerebral Hypoperfusion rats. CNS Neurol Disord Drug Targets. (2017) 16:1111–9. doi: 10.2174/1871527316666171026114043 29076436

[5] Lee K-S Kim J-S Kim J-W Lee WY Jeon BS Kim D. A multicenter randomized crossover multiple-dose comparison study of arotinolol and propranolol in essential tremor. Parkinsonism Relat Disord. (2003) 9:341–7. doi: 10.1016/S1353-8020(03)00029-4, PMID: 12853233

[6] Lee DB Woo YS Bahk WM. Use of Arotinolol pharmacotherapy to treat drug-induced tremor: a report of three cases. Pharmacopsychiatry. (2015) 48:176–8. doi: 10.1055/s-0035-1549931 25970026

[7] Meier T Buyse G. Idebenone: an emerging therapy for Friedreich ataxia. J Neurol. (2009) 256:25–30. doi: 10.1007/s00415-009-1005-0, PMID: 19283347

[8] Pradhan N Singh C Singh A. Coenzyme Q10 a mitochondrial restorer for various brain disorders. Naunyn Schmiedeberg's Arch Pharmacol. (2021) 394:2197–222. doi: 10.1007/s00210-021-02161-8, PMID: 34596729

[9] Wang Y Wu H Han Z Sheng H Wu Y Wang Y. Guhong injection promotes post-stroke functional recovery via attenuating cortical inflammation and apoptosis in subacute stage of ischemic stroke. Phytomedicine. (2022) 99:154034. doi: 10.1016/j.phymed.2022.154034, PMID: 35276592

[10] Fahn S Tolosa E Marín C. Clinical rating scale for tremor. Parkinson’s Dis Movement Disord. (1993) 2:271–80.

[11] Graham HK Rosenbaum P Paneth N Dan B Lin JP Damiano DL. Cerebral palsy. Disease Primers. (2016) 2:15082. doi: 10.1038/nrdp.2015.82, PMID: 27188686 PMC9619297

[12] Te Velde A Morgan C Novak I Tantsis E Badawi N. Early diagnosis and classification of cerebral palsy: an historical perspective and barriers to an early diagnosis. J Clin Med. (2019) 8:1599. doi: 10.3390/jcm8101599 31623303 PMC6832653

[13] Beaulieu-Boire I Aquino CC Fasano A Poon YY Fallis M Lang AE. Deep brain stimulation in rare inherited Dystonias. Brain Stimul. (2016) 9:905–10. doi: 10.1016/j.brs.2016.07.009, PMID: 27743838

[14] Wolf ME Blahak C Saryyeva A Schrader C Krauss JK. Deep brain stimulation for dystonia-choreoathetosis in cerebral palsy: Pallidal versus thalamic stimulation. Parkinsonism Relat Disord. (2019) 63:209–12. doi: 10.1016/j.parkreldis.2019.01.029 30718219

[15] Vidailhet M Yelnik J Lagrange C Fraix V Grabli D Thobois S. Bilateral pallidal deep brain stimulation for the treatment of patients with dystonia-choreoathetosis cerebral palsy: a prospective pilot study. Lancet Neurol. (2009) 8:709–17. doi: 10.1016/S1474-4422(09)70151-6, PMID: 19576854

[16] Krauss JK Loher TJ Weigel R Capelle HH Weber S Burgunder JM. Chronic stimulation of the globus pallidus internus for treatment of non-dYT1 generalized dystonia and choreoathetosis: 2-year follow up. J Neurosurg. (2003) 98:785–92. doi: 10.3171/jns.2003.98.4.0785, PMID: 12691403

[17] Labiano-Fontcuberta A Benito-León J. Essential tremor: update. Med Clin. (2013) 140:128–33. doi: 10.1016/j.medcli.2012.07.005, PMID: 22995841

[18] Teixeira MJ Cury RG Galhardoni R Barboza VR Brunoni AR Alho E. Deep brain stimulation of the dentate nucleus improves cerebellar ataxia after cerebellar stroke. Neurology. (2015) 85:2075–6. doi: 10.1212/WNL.0000000000002204, PMID: 26644050

[19] Bax M Tydeman C Flodmark O. Clinical and MRI correlates of cerebral palsy: the European cerebral palsy study. JAMA. (2006) 296:1602–8. doi: 10.1001/jama.296.13.1602 17018805

[20] Pelzer EA Hintzen A Goldau M von Cramon DY Timmermann L Tittgemeyer M. Cerebellar networks with basal ganglia: feasibility for tracking cerebello-pallidal and subthalamo-cerebellar projections in the human brain. Eur J Neurosci. (2013) 38:3106–14. doi: 10.1111/ejn.12314 23879686

[21] Fenoy AJ Schiess MC. Deep brain stimulation of the Dentato-Rubro-thalamic tract: outcomes of direct targeting for tremor. Neuromodulation. (2017) 20:429–36. doi: 10.1111/ner.12585 28256785

[22] Bostan AC Strick PL. The basal ganglia and the cerebellum: nodes in an integrated network. Nat Rev Neurosci. (2018) 19:338–50. doi: 10.1038/s41583-018-0002-7, PMID: 29643480 PMC6503669

